# Reactive Cu^2+^-peptide intermediates revealed by kinetic studies gain relevance by matching time windows in copper metallomics

**DOI:** 10.1093/mtomcs/mfad007

**Published:** 2023-02-14

**Authors:** Radosław Kotuniak, Wojciech Bal

**Affiliations:** Institute of Biochemistry and Biophysics, Polish Academy of Sciences, Pawińskiego 5a, 02-106 Warsaw, Poland; Institute of Biochemistry and Biophysics, Polish Academy of Sciences, Pawińskiego 5a, 02-106 Warsaw, Poland

**Keywords:** copper, kinetics, reactive intermediates, ATCUN motif, biological cycles

## Abstract

The purpose of this essay is to propose that metallomic studies in the area of extracellular copper transport are incomplete without the explicit consideration of kinetics of Cu^2+^ion binding and exchange reactions. The kinetic data should be interpreted in the context of time constraints imposed by specific physiological processes. Examples from experimental studies of Cu^2+^ ion interactions with amino-terminal copper and nickel binding site/N-terminal site motifs are used to demonstrate that duration and periodicity of such processes as bloodstream transport or neurotransmission promote the reaction intermediates to the role of physiological effectors. The unexpectedly long lifetimes of intermediate complexes lead to their accumulation and novel reactivities. The emerging ideas are discussed in the context of other research areas in metallomics.

## Graphical Abstract

**Graphical Abstract:**
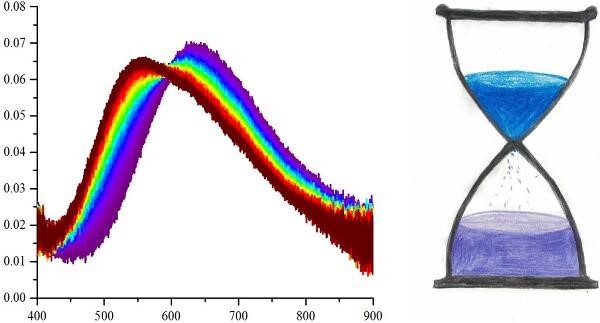
Studies of kinetics of Cu^2+^ binding to ATCUN/NTS motifs revealed reactive intermediates exhibiting lifetimes that matched the characteristic time windows relevant for copper physiology. This finding inspired a broader analysis, concluded with the notion that further progress in copper metallomics warrants the explicit consideration of rates and mechanisms of reactions.

## Introduction

The second half of the 20th century witnessed the nascent and huge developments of bioinorganic chemistry. Its systemic daughter, metallomics, emerged around the advent of the new century. Both remain on the trail of success, but we note that metallomics, a more catchy word gradually replaces and includes bioinorganic chemistry, despite the initial distinction. We will hence call them collectively ‘metallomics’ throughout the essay. This feat has been achieved by gradual adoption of spectroscopic and thermodynamic methods developed by inorganic and physical chemistry, enhanced by separation and identification techniques stemming from biochemistry and molecular biology. This interdisciplinary approach yielded very accurate structural description of metalloproteins, metal ion complexes with nucleic acids, such as ribozymes, and of complexes with small biological molecules, e.g. peptides or synthetic drugs. In many cases, quantitative information about the strength of metal ion interaction with the bioligand was obtained in the form of conditional or absolute stability constants. Multifactorial analyses and multidimensional imaging methods helped understand many physiological and toxicological processes exerted by metal ions on the level of tissues, organs, and organisms.^1^ All these advances included copper metallomics, subdivided into the extracellular and intracellular branches. In this short essay, we would like to propose that the time has come for (at least) extracellular copper metallomics to embrace another dimension, time. By doing so, one can produce testable hypotheses leading to a deeper understanding of copper physiology. The insights gained may also find application in other areas of metallomics.

## Steady-state and kinetic studies

The dominant role of equilibrium (steady-state) studies is in many instances dictated by inherently low sensitivity of a given technique, which requires lengthy accumulation of weak signal transients, e.g. in X-ray absorption spectroscopy or nuclear magnetic resonance. In thermodynamic techniques based on titrations, e.g. potentiometry or calorimetry, the correct quantitation of the signal requires achieving the state of equilibrium after adding each portion of the titrant. Solid-state structural techniques, like roentgenography, small-angle X-ray scattering, or cryoelectron microscopy, require not only the isolation of the studied (macro)molecule but also its immobilization.^2–4^

As a result, one obtains a static view of the interacting system at some sort of equilibrium. However, it is enforced by the set of conditions imposed by the experimenter rather than reflecting its actual participation in a broader homeostatic network. In many cases, it is sufficient and fruitful. Take a copper enzyme molecule isolated from the native organism or obtained by molecular biology techniques. Its test tube activity in response to various factors can be compared to its performance in its natural environment. Such studies are the essence of contemporary metallobiochemistry.

In quest of reconstructing the broader homeostatic picture, one could theoretically resort to a top-down approach, consisting of fractionation of the cell material and then identification/quantitation of metalloproteins and other metal ligands by electrospray or matrix assisted laser desorption/ionization mass spectrometry combined with inductively coupled plasma mass spectrometry. Unfortunately, Cu(II) ions are not sufficiently inert in terms of exchange reactions to perform such studies without risking artifacts, as for example demonstrated recently for ESI-MS experiments on Cu(II) peptide complexes.^5^

Alternatively, one could take a bottom-up approach and assume that the metal ion distribution in a biological compartment, e.g. blood serum, is governed by affinity constants that can be determined experimentally *in vitro*. It would then be sufficient to identify relevant components, measure all affinity constants for complexes of these components, and reconstruct the system computationally. The first stage is usually the most problematic one, but in principle, the substantial amount of required experimental work seems to be the main obstacle in achieving the aim. In technically difficult cases, such as large and fragile or poorly soluble biomolecules, their binding sites can be reconstructed using suitably designed simpler models. Such approach can be even more successful when aided by biological experiments, like silencing of a putative metabolic component in cells or knock-out of a protein in question.

The temporal aspect of copper metallomics has been mostly limited to metalloenzymatic catalysis and adventitious reactivity of complexes yielding toxic reactive oxygen species (ROS). Suitable examples are provided by studies of the role of Cu, Zn suproxide dismutase in amyotropic lateral sclerosis,^6^ and of contribution of cupric complexes of Aβ peptides to the Alzheimer's Disease (AD) pathology.^7^ The pioneering studies of Cu^2+^ binding to Aβ peptides by Hemmingsen and Ying laboratories serve as a notable exclusion, as they focused on the Cu^2+^ ion as the subject of the kinetic process rather than its catalyst.^8–12^

One area of copper metallomics that seems to be particularly suited for such kinetic studies is the Cu(I) transport in eukaryotic cells by a network of chaperones. The relays from the Ctr1 transporter via the chaperones towards the homeostatic enzymes, cytochrome c oxidase and superoxide dismutase, were reported to be occurring along the thermodynamic stability gradient.^13^ We are not aware, however, of specific kinetic studies of these relays, except of the general notion that they occur via the associative mechanisms.^14^,^15^ The Cu^+^ ion transport across the transmembrane channel(s) can also be considered as a series of ligand exchange reactions in its first coordination sphere. However, only the overall kinetics of this process was estimated in a cell culture study.^16^

Altogether, it seems that important temporal aspects of copper biology have not been addressed with sufficient scrutiny. This leaves an open question whether steady-state studies alone can provide a comprehensive view on copper physiology. We believe that the results of kinetic studies mentioned above suffice to make the case for the necessity of considering the timescale of chemical reactions involved in the transport of Cu^2+^, and perhaps also the Cu^+^ ion. This issue is elaborated in the next part of this paper, with the inclusion of our current experimental results.

## The biological venues for Cu(II) kinetics

Biology is all about change. The life cycle of a given organism is composed of component cycles, which have their characteristic time scales. In single-cell organisms, the main clock is provided by cell division. In complex multicellular organisms, there are many periodicities specific to the whole body, its organs, and tissues. Due to compartmentalization of their physiology into subcellular structures, cells, cavities, and vessels, these periodicities are associated with characteristic volumes of chemical reactions which underlie them. Examples of time and space constraints relevant for this discussion are given in Table 1. The emergent effect of compartmentalization on kinetics of Cu^2+^ reactions was covered elsewhere.^17^,^18^

**Table 1:** Examples of characteristic times and volumes of physiological processes related to copper physiology: human body, unless indicated otherwise.

Process	Periodicity	Volume	References
Neurotransmission across the synaptic cleft^a^	0.1–1 s	2–20 al	^19^
Saliva exchange (swallowing rate)	1 s	1 ml	^20^
Breathing/lung alveolar mucus processes	4–5 s	16 pl^b^	^21^,^22^
Blood circulation	1 min^c^	5 l	^23^
Cerebrospinal fluid exchange	6 h	30/200 ml^d^	^24^

^a^Glutamatergic synapses under various excitation modes.

^b^Calculated from the alveolar dimensions and mucus thickness estimates.

^c^The time in which a given blood voxel returns to a given point in the system, estimated on the basis of the cardiac output (heart stroke volume times heartbeats per minute).

^d^Ventricles/total.

The most important feature of homeostatic processes, such as those listed in Table 1, is the necessary cyclic reproducibility of initial/final and transient conditions for chemical reactions in their course. This means that whatever chemical or physical parameters, such as pH, concentrations of chemical species, electrostatic potential, surface tension, viscosity, etc., change during these processes, they must assume the same or very similar values at given time points of the process. In the bloodstream, the change has additional aspects of visiting various tissues in the body, combined with significantly different flow rates in arteries, capillaries, and veins.^25^ Therefore, the chemical reactions depending on these periodicities, or constituting them, must occur within specific timeframes and additionally in specific volumes. Below, we explore the emerging relevance of these factors for metallomics of Cu(II) transport in blood and in the brain. For the latter, we discuss the synaptic cleft and the cerebrospinal fluid (CSF).

## Copper trafficking in blood and brain

Despite the many years of research and debate, the issue of copper transport in blood is surprisingly far from a consensus. A number of studies of human, mouse, and rat blood serum indicated the presence of three major copper-binding proteins. Ceruloplasmin (Cp) provides the largest Cu(II) pool, estimated as ∼70% or more of total blood serum copper, but being an enzyme (ferroxidase), it does not exchange Cu(II) ions with other blood molecules.^26–32^ The remaining Cu(II) pool was reported to be divided between human serum albumin (HSA) and α2-Macroglobulin (α2M),^33^,^34^ complemented by a tiny low molecular-weight fraction, labelled small copper carriers (SCCs) by some researchers.^29^,^30^,^35^ However, two more recent studies indicated that no Cu(II) was associated with α2M fractionated from human blood,^27^,^36^ leaving HSA as a major candidate for distributing Cu(II) ions over the human (or rodent) body. This finding also agrees with the thermodynamic analysis presented by us recently.^37^ We demonstrated a direct contradiction between the reported total concentrations of HSA and α2M in blood serum, the reported Cu(II) affinities to these proteins, and the Cu(II) load of these proteins. In brief, α2M cannot bind the amount of Cu(II) ions similar to that at HSA and have a similar Cu(II) affinity when its molar concentration is 40–50 times lower.

HSA is a universal carrier for metabolites and drugs.^38^,^39^ In addition to Cu(II) ions, it also binds numerous essential and toxic metal ions at a variety of binding sites.^40^ The high affinity Cu(II) site is located at the N-terminus of HSA and comprises its first three amino acid residues, Asp-Ala-His. This sequence and its analogues substituted in positions 1 and 2 are collectively named amino-terminal copper and nickel binding site (ATCUN) motif,^41^ or alternatively N-terminal site (NTS).^42^ The *K*_d_ value of 100 fM was determined at pH 7.4, characteristic for blood serum.^43^ The low-affinity site is present in an interdomain pocket [multibinding site (MBS)] and comprises side chains of two His residues (H67 and H247).^42^ Its affinity is in the nanomolar range.^44^ The same site has been confirmed as a physiological Zn(II) carrier.^45^,^46^

The SCC fraction was discovered by size exclusion chromatography of blood serum, but has not been characterized so far with a satisfactory resolution. In the latest study, it was considered as a single chemical species with the molecular weight <1000 Da.^30^ This fraction in healthy people contains 0.2–2.5% of total Cu(II), depending on the report.^27^,^35^,^47^ Other reports proposed SCC to be a mixture of amino acids and short peptides. The amino acid histidine is its most abundant high affinity (nM or stronger) ligand, at 135 μM, and was inferred to constitute SCC as a leading component in a set of ternary complexes.^27^

Several peptides present in blood have higher Cu(II) affinities. The tripeptide Gly-His-Lys, a tissue hormone stimulating cell proliferation and wound healing, was isolated from blood as a Cu(II) complex.^48^,^49^ Its *K*_d_ value at pH 7.4 is 240 fM.^50^ Hepcidin (Hpc), a 25-amino-acid iron regulatory hormone with the ATCUN/NTS motif Asp-Thr-His, forms a much stronger complex, with *K*_d_ of 2.2 fM.^51^ Other peptides and proteins with similar femtomolar Cu(II) affinities are also present continuously or transiently in the bloodstream,^52^ and Cu(II) may be involved in their biological activity. However, with blood serum concentrations in the nanomolar range, their actual participation in the Cu(II) transport and distribution remains to be demonstrated. Altogether, the Cu(II) ion is probably sufficiently labile kinetically to scramble the native distribution during blood serum fractionation.^53–55^ Unfortunately, there are no experimental methodologies to determine nanomolar concentrations of Cu(II) complexes in blood serum directly.^56^ The prevailing opinion is that the full distribution of Cu(II) ions in blood serum could rather be reconstructed computationally from the affinity constants individually determined in the test tube, aided by crude blood serum fractionations into proteins and SCC. But even this approach may be more difficult than expected because of the formation of ternary complexes involving HSA and SCC members.^57^ Moreover, Cu(II) ions from such complexes can be assigned experimentally to either fraction, depending on technical details.^53^

Cu(II) ions are also present in synaptic clefts of glutamate-dependent neuronal networks, participating in the neurotransmission. The synapses in these networks transmit the signal by co-releasing glutamate and Zn(II) ions from presynaptic vesicles, while copper is released post-synaptically.^58^ Both the role of copper in this process and its chemical form are unknown. Histidine and glutathione were proposed as co-release ligands, stipulating either Cu(II) or Cu(I) redox state, respectively, but without a firm evidence.^59^,^60^ The peak copper concentration in this process as high as several hundreds of μM was reported.^61–63^

In human body, copper is transported across the biological membranes primarily by dedicated channels. The cells import copper passively using the Ctr1 transporter and export it actively using ATP-ases ATP7A or ATP7B, depending on the tissue.^64^,^65^ Loss-of-function mutations in Ctr1 are embryonic lethal, as demonstrated in mice,^66^ except of a very recently identified case that is associated with a significant developmental retardation.^67^ The mutations in ATP7A and ATP7B cause severe copper metabolism disorders in the form of Menkes (copper deficit) and Wilson (copper excess) diseases.^68^ Auxilliary tissue-specific copper transport alternatives probably exist, but have not been sufficiently characterized to be discussed here. All Ctr family members (also including Ctr2 and Ctr4) and the mentioned ATP-ases transport copper by their transmembrane channels solely as Cu^+^ ions.^69^,^70^ This is compatible with the intracellular transport of copper, which is conducted by Cu(I)-specific chaperones, but requires specific processes of Cu(II) reduction to Cu(I) prior to the copper acquisition from body fluids and, conversely, Cu(I) oxidation to Cu(II) after the release to these fluids. The latter issue, in particular, does not appear to have been researched. One can speculate, however, on the kinetic basis that Cu(I) oxidation to Cu(II) at the receptor mouth might occur during the Cu^+^ ion release or shortly afterwards in the bloodstream.^71^ Anyway, there are no reports on the Cu(I) presence in blood serum. Furthermore, the ATCUN/NTS site is present in the extracellular domain of Ctr1 proteins of most, if not all, mammalian species, suggesting that Ctr1 may expect Cu(II) rather than Cu(I) ions coming in.^72^,^73^ In contrast, Ctr1 variants in simpler organisms, like fungi, have short extracellular domains without Cu(II) binding sites and receive Cu^+^ ions from dedicated reductases.^74^

## Studies of kinetics of Cu^2+^ interactions with ATCUN/NTS motifs

Bearing this all in mind, we attempted to find out what are the rates of Cu^2+^ ion acquisition by ATCUN/NTS motifs and its release/relay to other chelators. We first studied the Cu(II) exchange between the ATCUN/NTS complexes. We found that Cu(II)-HSA yielded the Cu(II) ion to ATCUN/NTS sequences of Hpc_1__–__6_ and hCtr1_1__–__14_ peptides, representing the binding sites of Hpc and hCtr1, respectively. Both reactions followed the pseudo-first order kinetics, with similar *t*_½_ of ∼15 min.^51^,^75^ Analogous experiments were performed using the Cu(II)Aβ_4__–__16_ complex, using hCtr1_1__–__14_ and ethylenediaminetetraacetic acid (EDTA) as a Cu^2+^ ion recipients (see Table 2 for sequences of the relevant Aβ and hCtr1 peptides).^76^,^77^ Aβ_4__–__16_ was used as a model for the Aβ_4__–__42_ peptide, an abundant species of the Aβ family, that is likely to participate in the brain copper homeostasis.^78^ Aβ_4__–__42_ was recently confirmed to be the Cu(II) chelator in CSF.^79^ The *t*_½_ of several hours was obtained in both cases. Using the technically simpler Aβ_4__–__16_/EDTA system, we looked for agents that could accelerate such transfers, but we found only very moderate effects for non-redox agents.^80^ A more significant acceleration was achieved in the presence of thiol reductants, but still the reaction rates in the minutes range were seen.^81^ These results cast doubt on the competence of ATCUN/NTS motifs (or at least of that in Aβ_4__–_*_x_* peptides) in the Cu(II) exchange under physiological conditions.

**Table 2:** Sequences of main Aβ_1__–_*_x_* (not ATCUN/NTS), Aβ_4__–_*_x_* (ATCUN/NTS) peptides, and other ATCUN/NTS representatives—HSA, Hpc_1__–__6_, and hCtr1_1__–_*_x_*.^78^ ATCUN/NTS motifs are marked bold.

Peptide abbreviation	Sequence
Aβ_1__–__42_	DAEFRHDSGYEVHHQKLVFFAEDVGSNKGAIIGLMVGGVVIA
Aβ_1__–__40_	DAEFRHDSGYEVHHQKLVFFAEDVGSNKGAIIGLMVGGVV
Aβ_4__–__42_	**FRH**DSGYEVHHQKLVFFAEDVGSNKGAIIGLMVGGVVIA
Aβ_1__–__16_	DAEFRHDSGYEVHHQK
Aβ_4__–__16_	**FRH**DSGYEVHHQK
hCtr1_1__–__14_	**MDH**SHHMGMSYMDS
hCtr1_1__–__3_	**MDH**
HSA	**DAH**
Hpc_1__–__6_	**DTHFPI**

**Fig. 1:**
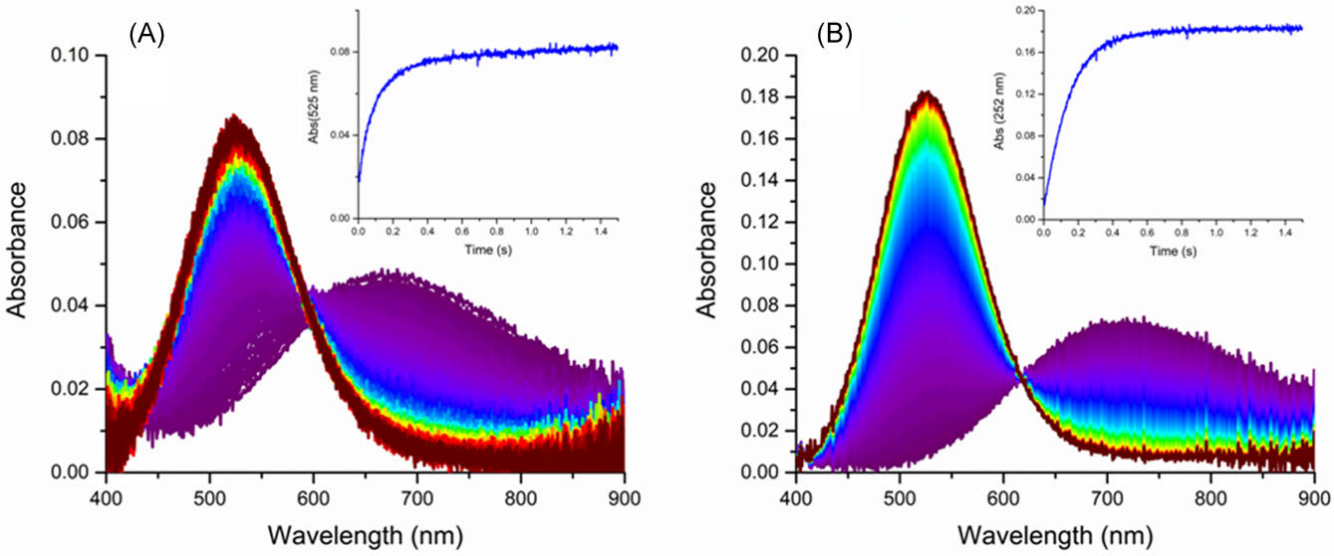
The examples of reactions of Cu^2+^ ions with ATCUN/NTS peptides recorded by the stopped-flow technique with diode array spectral detection. The apparatus dead time was 2 ms. The insets present the kinetic traces at 525 nm (the ATCUN/NTS 4N complex formation). (A) The reaction of 1 mM Aβ_4__–__16_ with 0.9 mM Cu^2+^ ions at pH 7.4, 200 mM phosphate buffer (P. Szczerba, R. Kotuniak, and W. Bal, manuscript in preparation). (B) The reaction of 2 mM Gly-Gly-His with 1.8 mM Cu^2+^ ions at pH 6.0 (200 mM MES buffer).^85^ The *t*_½_ for the IC transition to 4N were 95 and 97 ms, respectively.

**Fig. 2:**
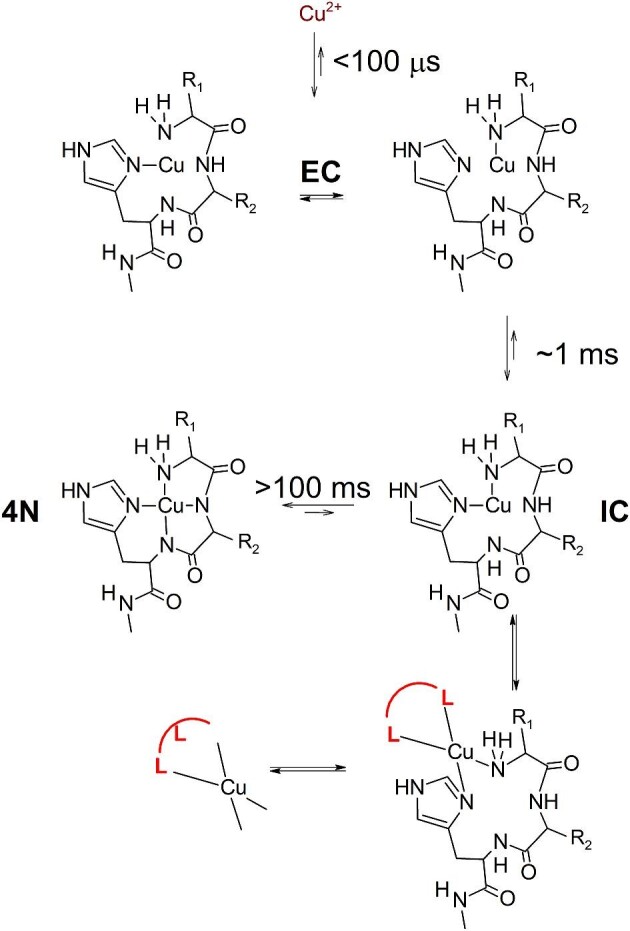
The reaction scheme for stepwise Cu^2+^ binding to ATCUN/NTS motifs. Reaction half times for individual reaction steps are provided. The EC, IC, and 4N complexes were present in all peptides studied so far.^37^,^84–86^ The ‘unproductive conformers’ label regards the arrangements of the peptide main chain, which cannot adopt the tight loop structure of the 4N complex (as shown in Supplementary Fig. S1 in reference [85]). Charges of complexes and coordinated water molecules in the Cu^2+^ ion, EC, and IC were omitted for clarity. The unsymmetrical double arrows indicate the direction in which the equilibrium is shifted in a qualitative manner.

Aiming at a better understanding of mechanism of these Cu(II) exchange reactions, we performed fast kinetics studies of the binding of the Cu^2+^ ion to ATCUN/NTS peptides. The reaction times longer than 1.5–2 ms were followed by stopped-flow systems equipped with diode array detectors in the visible spectral range. Shorter reaction times, down to ∼100 μs, were probed using the Microsecond Freeze-HyperQuenching (MHQ) technique, where the Cu(II) species were detected using frozen solution mode EPR measurements of the individually collected samples.^82^,^83^ The initial stopped-flow and all MHQ experiments were performed in the laboratory of Professor Peter-Leon Hagedoorn at Delft University of Technology. Further stopped-flow studies were performed both in Delft and in our home laboratory. The technical ability of direct identification of coordination modes of Cu(II) ions were crucial for identifying the reaction intermediates. Two examples of such stopped-flow experiments are provided in Fig. 1. In the first study, the Gly-Gly-His tripeptide was used as the simplest possible ATCUN/NTS motif. Surprisingly long lifetimes of reaction intermediates were observed, and intramolecular rearrangements were proposed to be their source.^84^ The reaction scheme based on this study and the follow-up work is provided in Fig. 2.^37^,^84^,^85^ The *t*_½_ of the intramolecular rearrangement of the more stable reaction intermediate [intermediate complex (IC) in Fig. 1] to the final standard ATCUN/NTS complex [four nitrogen (4N)] was ∼100 ms under the applied experimental conditions. The IC was confirmed by the steady-state UV/Vis experiment. The same reaction mechanism and similar reaction lifetimes were observed for the Aβ_4__–__16_ peptide, on a highly sensitive stopped-flow system, detecting the fluorescently labelled peptide.^77^ The systematic follow-up study indicated that peptides with larger side chains in positions 1 and 2 often exhibited much longer *t*_½_, even exceeding one second.^85^ Yet slower reactions were found for peptides containing additional Cu(II)-binding sites, which offer alternative molecular arrangements for IC species.^86^ The extreme case of reaction retardation was detected for HSA, with *t*_½_ approaching 10 s.^87^

These results have interesting consequences. Long lifetimes of IC reaction intermediates in Cu^2+^ binding to ATCUN/NTS motifs are due to an intramolecular rearrangement mechanism. This means that these lifetimes can be applied to model the behaviour of individual proteins/peptides at their biological concentrations. Furthermore, both IC and early complex (EC) are steady-state reaction products, minor at physiological pH but abundant at lower pH values.^84^,^85^ Hence, all reaction steps in Fig. 2 are reversible, ruled by the mass action law. These observations find confirmation in the data on Cu(II) exchange for HSA and Aβ_4__–__16_, indicating that the rates of these reactions depend primarily on the properties of the Cu(II) donor.^51^,^75–77^ On the other hand, the complicated concentration dependence of the action of EDTA on Aβ_4__–__16_ indicates the active role of the former in the transfer reaction.^77^ Furthermore, the rate of copper release to metallothionein can be significantly accelerated by thiol reductants, much more active in this respect that ascorbate.^81^,^88^ It can also be somewhat accelerated by non-redox external factors.^81^,^89^

**Fig. 3:**
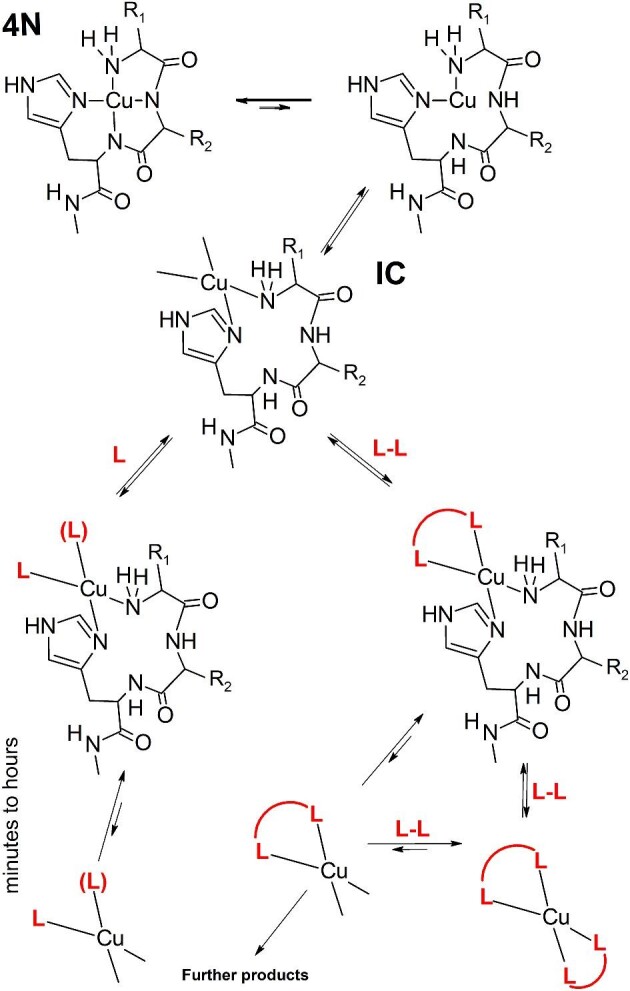
The scheme for further reactions of EC and IC based on experiments with added chelators. L is a monodentate ligand, (L) means an optional second molecule in the complex, and L–L is a chelating ligand. Coordinated water molecules and complex charges were omitted for clarity. The unsymmetrical double arrows indicate the direction in which the equilibrium is shifted in a qualitative manner.

**Fig. 4:**
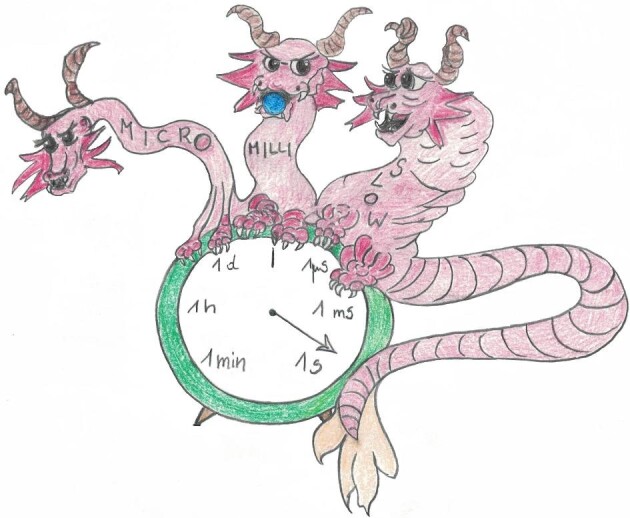
The allegoric representation of the ATCUN/NTS interactions with the Cu(II) ion. Three heads of the ATCUN/NTS dragon sitting on the reaction clock, Micro, Milli and Slow, represent the three Cu(II) coordination states: EC, IC, and 4N, depicted in the molecular detail in Fig. 2. In the ms range of reaction times, Micro has done its job and stays away, while Milli, holding copper (the blue ball), peeks at Slow, who is eager to intercept it and stay with it in the endless state of equilibrium. Milli, however, is free to do other things, as indicated in Fig. 3.

This complicated set of results can be rationalized on the basis of features emerging from the established reaction mechanism (Fig. 2) and considerations based on competition and Cu(II) exchange reactions (Fig. 3). First, there is a bimolecular reaction between the Cu^2+^ ion and the ATCUN/NTS peptide, followed by a series of IC intramolecular arrangements. All steps of this mechanism are reversible, as evidenced by the detection of EC and IC species at lower pH values, paving the way to the dissociative mechanism of Cu^2+^ ion exchange. However, the partially unoccupied Cu(II) coordination spheres in EC and IC are prone to formation of ternary complexes with monodentate and chelating ligands, depicted as L and L–L, respectively, in Fig. 3. Conversely, the Cu(II) ion may be delivered to ATCUN/NTS by such ligands, with significant effects on reaction rates (R. Kotuniak and W. Bal, manuscript in preparation). Only the multistep associative mechanism, such as the one sketched at the bottom of Fig. 3, can explain the concentration/rate relationship of Cu(II) transfer from Aβ_4__–__16_ to EDTA.^77^ The particular slowness of the Cu(II) exchange between two ATCUN/NTS peptides may be due to a low abundance of IC (end extremely low abundance of EC) in one both partners.^76^ Alternatively, the ternary intermediate between them may be hindered sterically. The contrastingly fast release of Cu(II) by HSA (with K_d_ very similar to that of Ctr1_1__–__14_) may be a sign of self-catalysis in a large protein, including reactive side chains on the protein surface.^43^,^57^,^75^ Much more research is required to sort these issues out. However, with the data available already, we can tentatively state that the Cu(II) release from ATCUN/NTS has a largely associative character. The ultimate proof for this statement will be provided by measurements of (as much as possible) unassisted *K*_d_ of ATCUN/NTS complexes, currently planned in our laboratory. For now, we can safely propose that each cupric ATCUN/NTS complex has a characteristic time scale of Cu^2+^ binding and release/exchange reactions. This is illustrated in an artistic fashion in Fig. 4 and can be now used to assess the relevance of specific ATCUN/NTS complexes in Cu(II) physiology by taking into account the time constraints of individual processes.

## From the timing of reaction stages to creating and selecting physiological hypotheses

Let us start by considering how copper is acquired from food. The copper requirement of adult humans is between 0.8 and 2.4 mg/d.^90^ Copper ions mobilized during digestion are collected from the lumen of small intestine by enterocytes using hCtr1, followed by their export by ATP7A to blood.^91^ Copper ions are then carried by portal vein to liver, its main target organ, where they are collected by hepatocytes for the Cp holoenzyme assembly. The excess of copper is secreted by liver into bile by the action of ATP7B.^91^,^92^ The uncollected leftover is carried away by the hepatic vein to be distributed to other tissues. The anatomy of the portal vein is critical for this analysis. Its length is ∼30 cm, and the flow rate through it varies between 10 and 30 cm/s in healthy adults.^93^ This means that it takes several seconds, perhaps as little as one, for copper ions to reach liver. Our current experiments indicate that this time is not sufficient for significant amounts of copper collected from small intestine to be bound at the ATCUN/NTS site of HSA (*t*_½_ ∼10 s, to which the unknown time required for Cu(I) oxidation to Cu(II) should be added, as prerequisite of the ATCUN/NTS complex formation). Hence, opposite to common views, ATCUN/NTS motifs of HSA and other proteins/peptides cannot deliver copper from small intestine to liver. They, however, might do so in other tissues because it takes about a minute for a given blood voxel and a given HSA molecule to make the full circulation loop.^23^ The *t*_½_ ∼15 min for Cu(II) release from HSA seems to be compatible with such a broad distribution function. In this way, the ATCUN/NTS Cu(II) complex of HSA, which circulates in the bloodstream at the concentration estimated between 2.4 and 4.2 µM, appears to represent a systemic copper store, rather than a delivery pool.

The time restriction for copper chemistry in the glutamate-dependent synapse is even more severe than in the portal vein. Under the resting conditions, these neurons fire about once per second (1 Hz).^94^ But this rate may increase >50-fold upon stimulation while maintaining the full signal reproducibility.^19^ This suggests that all copper released into the synaptic cleft must be removed from it between the firings, either by the action of hCtr1 and maybe other membrane transporters or by efflux towards the CSF. Aβ_4__–_*_x_* peptides (*x* = 40, 42) were suggested to act as Cu(II) scavengers supporting such function for their ability to quench adventitious binding and ROS generation by Cu(II).^78^,^88^,^95^ This concept was incomplete, however, because of the sluggishness of their ATCUN/NTS motif in Cu(II) release to the hCtr1_1__–__14_ model peptide.^76^ As mentioned above, experiments dedicated to finding agents that might catalyse/accelerate the Cu(II) transfer were not very successful.^80^,^81^,^89^ This contradiction seems to be now solved by the Cu^2+^ binding kinetics to the Aβ_4__–__16_ model, compared to the time scale of synaptic events. In the 20 ms, time window suggested by the stimulated firing trains of glutamatergic neurons, practically no 4N complex may be formed, whereas the IC species will be formed within one ms.^77^,^89^ This selectivity is supported by the peculiar geometry of the synaptic cleft, where the distance between the pre- and post-synaptic neurons is about 20 nm^96^ and the diffusion across it is of the order of single ms.^97^

In this way, compared with the physiological time constraint of neurotransmission, kinetic experiments indicated the IC as essentially the only possible Cu(II) complex of Aβ_4__–_*_x_* peptides during neurotransmission. In a strikingly close analogy to the emerging picture of Cu(II) interactions with albumin in blood, the Cu(II) complexes of Aβ_4__–__42_ and Aβ_4__–__40_ peptides were detected in the CSF by mass spectrometry.^79^ Only the 4N ATCUN/NTS complex is sufficiently inert in ESI-MS experimental conditions to be detected,^5^ and CSF is the drain for the synaptic fluid,^98^ hence Cu(II)/Aβ_4__–_*_x_* can migrate there from the neuronal pathways in the cortex and hippocampus, having enough time to form the kinetically inert 4N species in the process. As discussed before, such species are well suited for protecting the brain from copper-generated ROS.^78^

The predominance of kinetics over thermodynamics in the millisecond time scale may also vindicate complexes, which are considered too weak to participate in Cu(II) biology. One case is the relevance of Cu(II) interactions with Aβ_4__–_*_x_* versus Aβ_1__–_*_x_* peptides. The latter have been studied most commonly and were proposed to contribute to Alzheimer's disease pathology by generating deleterious ROS.^7^ However, they bind Cu(II) with *K*_d_ of 100 pM,^99^ more than ‘thousand-fold weaker than Aβ_4__–_*_x_* peptides. Accordingly, a benchtop experiment demonstrated that upon adding Aβ_4__–__16_ to the Cu(II)Aβ_1__–__16_ solution the Cu(II) ions were smoothly transferred to Cu(II)Aβ_4__–__16_ within the test tube mixing time of several tens of seconds.^95^ Now, within the new paradigm, and with the initial Cu(II) binding to Aβ_1__–__16_ measured under the same conditions to be 2.5 times faster than to Aβ_4__–__16_,^9^ one will have to consider both agents simultaneously in designing new experiments. These experiments should also reflect the stochastic nature of interactions in the synaptic cleft and similarly small biological compartments, enforced by low counts of interacting molecules.^17^,^18^

The long-living IC could also relay Cu(II) to similar transient species of other biological copper carriers based on their kinetic, rather than steady-state properties, according to the mechanism presented in Fig. 3. Furthermore, the IC exhibits a reversible Cu(II)/Cu(I) electrochemistry, providing pathways towards explaining the unsolved issue of reductive acquisition of copper by Ctr1. In the simplest scenario, the IC may be just the reduction site.^100^ Especially, the interactions with small Cu(II) binding molecules may be part of the mechanistic link between the aberrations of SCC fraction in blood and the incidence of AD.^101^,^102^

The single-second time windows apparent for saliva and lung mucus call for analogous investigations, as semi-stable reaction intermediates may gain importance at the expense of the stable species observed in previous equilibrium studies. In saliva, this may regard histatins, a family of ATCUN/NTS antimicrobial peptides that bind Cu(II) ions avidly.^103^ This interaction was evoked in proposing how histatins exert their protective function against infections in the mouth. Equipped with the new kinetic paradigm, one may start asking new scientific questions, such as what happens to these complexes in the digestive tract after the swallow and whether they can be active on the way.

Similar rules will probably apply to other metal ions and other bioligands, paving the way to novel metal ion binding and exchange interactions, to be revealed by future systematic studies. Among many areas of potential interest we would like to suggest the extracellular and intracellular Zn(II) trafficking, for example the mechanism and time course of Zn(II) release from insulin hexamers and its reabsorption by HSA, crucial for maintaining the insulin activity,^45^,^46^,^104^ and intracellular delivery of Zn(II) ions to thousands of zinc proteins by a heterogeneous system comprising transporters, chaperones, and small molecules.^105–108^

## Conclusions

The experimental determination of rate constants for multistep reactions of Cu(II) ion binding to biological ligands by fast mixing techniques has provided a unique view on reaction intermediates with lifetimes reaching into the seconds and minutes. A comparison of these lifetimes with the rhythms of physiologic clocks can be used as a powerful validation tool for molecular concepts in metallomics, e.g. disqualifying complex species that are too slow to be formed within the available time. The specific chemical reactivities of unexpected reaction intermediates may be crucial for copper transport phenomena, as they accumulate due to their long lifetimes. The proposed approach can also provide novel mechanistic ideas also for other metal ions. Such new concepts can be verified in appropriately designed time-dependent biological/analytical experiments.

## Data Availability

No new data were generated or analysed in support of this research.
